# Cat-to-Human Transmission of *Mycobacterium bovis*, United Kingdom

**DOI:** 10.3201/eid2512.190012

**Published:** 2019-12

**Authors:** Catherine M. O’Connor, Muhammad Abid, Amanda L. Walsh, Behrooz Behbod, Tony Roberts, Linda V. Booth, H. Lucy Thomas, Noel H. Smith, Eleftheria Palkopoulou, James Dale, Javier Nunez-Garcia, Dilys Morgan

**Affiliations:** Public Health England, London, UK (C.M. O’Connor, A.L. Walsh, H.L. Thomas, D. Morgan);; Public Health England, Chilton, UK (M. Abid, B. Behbod);; Animal and Plant Health Agency, Oxford, UK (T. Roberts);; Public Health England, Fareham, UK (L.V. Booth);; Animal and Plant Health Agency, Weybridge, UK (N.H. Smith, E. Palkopoulou, J. Dale, J. Nunez-Garcia)

**Keywords:** Mycobacterium bovis, bacteria, tuberculosis and other mycobacteria, cat, human, transmission, zoonoses, United Kingdom

## Abstract

Human infection with *Mycobacterium bovis* is reported infrequently in the United Kingdom. Most cases involve previous consumption of unpasteurized milk. We report a rare occurrence of 2 incidents of cat-to-human transmission of *M. bovis* during a cluster of infection in cats.

In the United Kingdom, *Mycobacterium bovis* infection in humans is relatively rare (*1*), and most cases involve previous exposure to well-recognized risk factors, such as unpasteurized milk (*2*). However, with >4,500 new cases reported in cattle herds each year during 2014–2018 (*3*), *M. bovis* remains a major issue for animal health in large parts of England and Wales.

Cats are considered spillover hosts of *M. bovis* in the United Kingdom. During 2002–2010, <30 feline cases of *M. bovis* infection were confirmed by laboratory culture each year by the Animal and Plant Health Agency *M. bovis* Reference Laboratory (*4*). Cases of *M. bovis* infection in cats are generally restricted to areas to which bovine tuberculosis (TB) is endemic (*5*), where infected cattle and wildlife have the potential to introduce *M. bovis* into cat populations. Most reported feline cases of *M. bovis* infection in the United Kingdom are sporadic, and outside the occasional household-linked cases, spatially or temporally linked clusters are unusual.

The potential for cat-to-human transmission of *M. bovis* has always been recognized. Although concurrent infection in cats and humans in the same household has been reported (*6*), and reports of potential transmission exist (*7*), documented transmission events have not been clearly described. We report a rare occurrence of microbiologically and genetically confirmed cat-to-human transmission of *M. bovis*.

## The Study

During December 2012–March 2013, a veterinary practice in Berkshire, England, diagnosed 7 confirmed (culture from lesions or wounds) and 2 suspected (clinically compatible) cases of *M. bovis* disease in domestic cats. One additional suspected case was identified after an interview with an affected household. No samples were available from any of the suspected cases for confirmation for this study. The 10 cats belonged to 9 separate households, of which 6 were <250 m from each other. All cats had severe systemic infection, including discharging lymph nodes, nonhealing or discharging infected wounds, and radiographic pulmonary signs. Isolates from the culture-confirmed cases were of the same genotype (10:u), were similar by whole-genome sequencing, and separated into 2 clusters by a single informative polymorphism (*8*). Veterinary investigations did not determine the source of infection, but the source was believed to be infected wildlife, most likely rodents or badgers, for at least some of the cats. Further information on the investigation into this cluster of infection in cats has been reported (*8*).

The unusual size and severity of the cluster of feline *M. bovis* cases led to the decision that TB screening (*9*) would be offered to all human household members and others who had close contact with the infected cats. Local Health Protection Teams of Public Health England identified 39 human contacts; 24 accepted TB screening. Three persons (person A, 13 years of age; person B, 18 years of age; and person C, 39 years of age) were positive for latent TB infection (LTBI) by a combination of interferon-γ release assays and Mantoux screening tests; none showed evidence of active disease.

These 3 persons with LTBI reported close contact with 2 cats with culture-confirmed *M. bovis* infection while the cats had clinical disease (1 had a discharging nonhealing wound and 1 had a discharging lymph node). Apart from contact with the infected cats, no other risk factors for *M. bovis* or *M. tuberculosis* exposure were identified. Because it was not possible to isolate the causative organism from cats or persons with LTBI or identify the likely exposure or source of infection, it was not possible to determine whether transmission of *M. bovis* from these affected cats to their human contacts had occurred. All 3 persons with LTBI were offered chemoprophylaxis, but only 1 (person A) accepted. All 3 persons were advised to go to local health services if any symptoms potentially indicative of active TB developed.

Six months after initial screening, person B was medically assessed because of nonspecific abdominal pain. Chest radiographs showed evidence of pathologic changes potentially indicative of TB. *M. bovis* was isolated from pleural biopsy samples. Shortly after person B had clinical illness, a nonhousehold human contact (person D, 20 years of age) of person B and their cat also had *M. bovis* isolated from pleural biopsy samples after reporting chest pain and fever. Person D had initially declined screening. Persons B and D had close contact with the infected cat while it was systemically ill (including a discharging wound). The cat died before *M. bovis* infection was diagnosed. Both persons completed a 9-month course of rifampin, isoniazid, pyrazinamide, and ethambutol and responded well to treatment (*10*).

Molecular analysis showed that persons B and D, who had active *M. bovis* disease, and the cat all had *M. bovis* isolates of the same genotype. Whole-genome sequencing of samples from one of the humans and the cat showed that their isolates were indistinguishable (Figure). (Sequencing was not possible for the isolate from the second human patient.) This evidence, coupled with the timeline of onset of disease in the cat (March 2013) and its human contacts (October 2013), and the lack of any other risk factors for exposure to *M. bovis*, indicated that the cat was the likely source of infection for these 2 affected persons.

**Figure:**
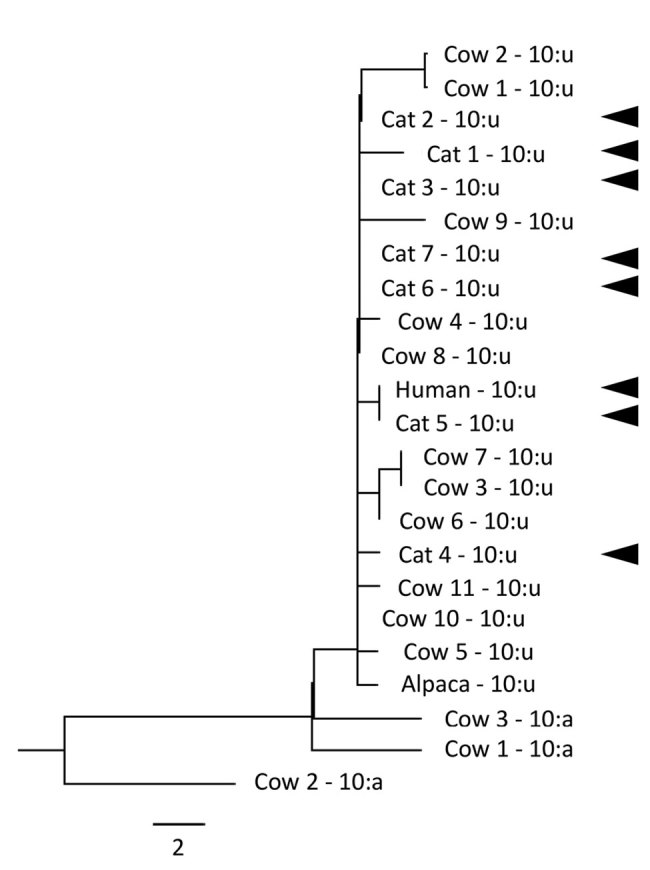
Whole-genome sequencing phylogenetic relationship of genotype 10:u isolates of *Mycobacterium bovis* from 1 human, 7 cats, 11 cattle, and 1 alpaca, and 10:a isolates from 3 cattle (maximum-likelihood tree of all single-nucleotide polymorphisms [SNPs]). Cat and human isolates are indicated by solid arrowheads. Heat-killed cultures were sequenced by using a MiSeq Sequencer (Illumina, https://www.illumina.com), and reads were mapped by using reference strain AF2122. The average coverage ranged from 23.9-fold to 88.8-fold. The human and their household cat contact (cat 5) isolates were indistinguishable in their genome sequences. Scale bar indicates SNPs.

## Conclusions

Before this incident, the absence of confirmed reports of human cases of *M. bovis* infection acquired from pet cats led us to believe that the risk for cat-to-human transmission was negligible. Thus, no public health action was warranted. However, with the evidence of transmission from 1 cat to these 2 patients, the risk for spread of *M. bovis* from cats to their human contacts was increased from negligible to low (*11*). Cats with clinical signs compatible with disseminated disease are believed to have the greatest risk to humans, most likely by ingestion from a contaminated environment, following handling of discharges from exudative tuberculous lesions, or by aerosols from cats with respiratory signs or aerosol-generating procedures.

Public Health England now advises that all close contacts of household companion animals with confirmed *M. bovis* infections should be assessed by a public health professional and receive guidance on how to best minimize zoonotic transmission (*12**,**13*). In addition, as part of an enhanced surveillance system in England and Wales, newly diagnosed human case-patients with *M. bovis* infection are now also asked explicitly about contact with pets with suspected or confirmed *M. bovis* disease (*14*).

In summary, *M. bovis* disease in companion animals, particularly cats with severe systemic features including exudative lesions, can no longer be regarded as posing a negligible public health risk. Guidance should be provided to minimize the risk for transmission to human contacts.

## References

[1] Public Health England. Tuberculosis caused by *Mycobacterium bovis* (*M. bovis*): notification data; 2018 [cited 2018 Nov 13]. https://www.gov.uk/government/publications/mycobacterium-bovis-mbovis-tuberculosis-annual-data

[2] Davidson JA, Loutet MG, O’Connor C, Kearns C, Smith RM, Lalor MK, et al. Epidemiology of *Mycobacterium bovis* Disease in Humans in England, Wales, and Northern Ireland, 2002-2014. Emerg Infect Dis. 2017;23:377–86. 10.3201/eid2303.161408 28220748 PMC5382737

[3] Department for Environment, Food, and Rural Affairs. Tuberculosis (TB) in cattle in Great Britain (update 17 October 2018); 2018 [cited 2018 Nov 13]. https://www.gov.uk/government/statistical-data-sets/tuberculosis-tb-in-cattle-in-great-britain

[4] Department for Environment, Food, and Rural Affairs. Incidents of *M. bovis* infection in non-bovine domestic animals and wild deer in GB confirmed by laboratory culture; 2015 [cited 2018 Dec 3]. www.gov.uk/government/uploads/system/uploads/attachment_data/file/232602/bovinetb-otherspecies-27aug13.xls

[5] Gunn-Moore DA, McFarland SE, Brewer JI, Crawshaw TR, Clifton-Hadley RS, Kovalik M, et al. Mycobacterial disease in cats in Great Britain: I. Culture results, geographical distribution and clinical presentation of 339 cases. J Feline Med Surg. 2011;13:934–44. 10.1016/j.jfms.2011.07.012 22079343 PMC10832973

[6] Ramdas KE, Lyashchenko KP, Greenwald R, Robbe-Austerman S, McManis C, Waters WR. *Mycobacterium bovis* infection in humans and cats in same household, Texas, USA, 2012. Emerg Infect Dis. 2015;21:480–3. 10.3201/eid2103.140715 25695666 PMC4344262

[7] Lewis-Jonsson J. Transmission of tuberculosis from cats to human beings. Acta Tuberc Pneumol Scand. 1946;20:102.

[8] Roberts T, O’Connor C, Nuñez-Garcia J, de la Rua-Domenech R, Smith NH. Unusual cluster of *Mycobacterium bovis* infection in cats. Vet Rec. 2014;174:326. 10.1136/vr.102457 24676263 PMC3995260

[9] Public Health England. Tuberculosis screening; 2018 [cited 2019 Jul 30]. https://www.gov.uk/guidance/tuberculosis-screening

[10] Talwar A, McGown A, Langham T, Abid M, Pilling J; Department of Respiratory Medicine, Royal Berkshire Hospital. A very strange tail. Thorax. 2014;69:1159–60. 10.1136/thoraxjnl-2014-206033 25335487

[11] Human Animal Infections and Risk Surveillance group (HAIRS). Qualitative assessment of the risk that cats infected with *Mycobacterium bovis* present to human health; 2014 [cited 2018 Dec 3]. https://www.gov.uk/government/publications/hairs-risk-assessment-mycobacterium-bovis-in-cats

[12] Public Health England. Bovine tuberculosis: guidance on management of the public health consequences of tuberculosis in cattle and other animals (England); 2014 [cited 2018 Dec 3]. https://www.gov.uk/government/publications/bovine-tuberculosis-tb-public-health-management

[13] UK Government. Bovine tuberculosis in domestic pets; 2014 [cited 2019 Jul 30]. https://assets.publishing.service.gov.uk/government/uploads/system/uploads/attachment_data/file/596240/AG-TBYP-01e.pdf

[14] Public Health England. *Mycobacterium bovis* (*M. bovis*): enhanced surveillance questionnaire; 2016 [cited 2018 Dec 3]. https://www.gov.uk/government/publications/mycobacterium-bovis-m-bovis-enhanced-surveillance-questionnaire

